# Supplementary material for: A case study of the features and holistic athlete impacts of a UK sports-friendly school: Student-athlete, coach and teacher perspectives
Source: PLoS One. 2022 Nov 30;17(11):e0278401. doi: 10.1371/journal.pone.0278401 (PMC9710759; doi:10.1371/journal.pone.0278401)
Supplement: S1 Dataset — (XLSX) [file pone.0278401.s001.xlsx]

**S1. Dataset. Summary of the holistic athlete impacts of a UK sports-friendly school**

| **High order theme** | **Middle order theme** | **Lower order theme** | **Examples of what stakeholders shared/raw data** |
| --- | --- | --- | --- |
| Academic & vocational impacts | Immediate - negatives | Difficult jump academically between GCSE and A-Level/BTEC | "As a boarder. It hit me hard. That first week was difficult. Like I would come in and crash and I would sleep for like 2 hours, then after the first week, you didn’t have time to sleep for two hours because you have stuff to do, you have work to do, you have to get your stuff ready for the next day, certain days you have assignments, certain research you have to do to get ahead. If it’s a lesson you don’t understand, because college in general is a big jump, you have to get ahead on that. So, I think that initial challenge was quite difficult." - A18 |
|  |  | Difficulty balancing the high academic workload with high volume of sport | "I think it was definitely a struggle at first to manage my time between my academics and my training." - A14 |
|  |  |  | "I think for me personally, because I came in on an academic and a sport scholar. I definitely feel that pressure in terms of being able to balancing both side of things, because obviously you want to pursue basketball, but you can’t let education drop. As one for first half term was very very difficult, because generally the jump between GCSE and college, is quite difficult. It was a new subject like. Like that pressure for me personally, I didn’t find that positive. " - A18 |
|  | Intermediate - positives | Academic security (e.g., GSCE and A-level qualifications) | "Other short-term positives I think is their ability to continue with their education and to get their A levels and to think about a plan B. Because if I look at other options that the students could take, you know, it might be that their main focus or 75 - 80 percent of their focus is football. 20 percent might be their academics. Whereas here, academics comes first. And the players and the students realise that. And they know that if they don't do their academic work, then they're gonna drop off the football. So, like...it's almost like an incentive for them to do well with their A levels and their academics. And when students come to look around the school and when we first meet their families, we say...we're open and honest with them and we say, you know, that academics comes first. And we talk about the realism in terms of the chances of your son, your daughter becoming a professional athlete. It's minimal. So, it's important. We're gonna provide an opportunity for your son or daughter to compete at a really good level, but ultimately, it's the A levels and the education that's probably gonna set you up for the rest of your life." - C3 |
|  | Intermediate - negatives | Lower academic grades/underperforming | "They’ve maybe underachieved in their first year, playing catch up a little bit, probably didn't get the grades that they should have got." – C3 |
|  | Long-term - positives | Dual options (professional sport route or higher education success) | "I say to them, this place will give you a foundation of where you want to go. Be that, you know, if you do, if you're flying in your sport, that could be down a professional route, or it could be down like the books League, through universities and things like that." – C6 |
|  |  | University sport scholarships | "You know we had...we had a student last year who's...who came for one year and he's gone onto to sign for a professional club. That's an amazing...an amazing story, students going on to...to play for top universities like Loughborough, unbelievable football programs and getting a really good education. And then we have other students that...that have gone over to America to get a good education, play in amazing universities with amazing facilities and just the life skills of, you know, three or four years in America." - C3 |
|  | Long-term - negatives | Second or third choice university | "Maybe didn't get the university that they were hoping for so they've had to settle for like a, you know, a second or third choice university because they couldn't quite... because they couldn't quite handle that transition and, again, I think...it comes down to personality, for me, in terms of dealing with workload and...and things like that. So that...that could be...that could be one." – C3 |
| Athletic & physical impacts | Intermediate - positives | Performance sport education | "I think also in terms of development as an athlete as well as person, to the general environment here, being surrounded by better players, more qualified staff, coaches, having that athlete structure, there are elements that you wouldn’t have considered like nutrition and your diet and stuff like, how much am I drinking, the type of stuff you eat. All of that, has definitely helped me realise, that if I am going to pursue this thing or go somewhere with this that it is a completely different ball game." - A18 |
|  |  |  | "I have done a lot of S&C and stuff before, but not to this standard. I like the fact it is a higher standard and have gained an understanding of why we are doing, rather than just doing it. I think it definitely helps with the mental side of sport, because I am actually understanding why I am doing something, rather than just doing it for no reason." – A6 |
|  |  | Performance development (technical, tactical, psychological and physical) | "I can see how much I’ve like improved from just being here five months than what I would have done if I never came here. S&C and then also like the [sport] sessions we’re doing with C1 as well. He like bases it on a lot of different things rather than one aspect." – A2 |
|  |  |  | "And I think this year, I’m getting to see more of the technical parts, like, okay, if he going there, I have to be there, and if he is going in, I have to go clear out. Which is something that I didn’t kind of understand last year, and it has really improved my game being part of the program." - A14 |
|  |  | Positive general health and well-being | "I think general health and wellbeing. You know, being active. Pushing their bodies to the maximum to improve themselves from a physical point of view. You know, the long-term physical benefits in terms of health and wellbeing. So, the wellbeing aspect of it because they’re performing, they’re being active on a daily basis. " – C3 |
|  |  |  | "I think just from the fitness aspect and the confidence that you try and give them within… the way that you work with them, I think that helps massively mentally. I think with their wellbeing and with, obviously with their physical fitness and their health, I think that has massive implications." – C5 |
|  | Intermediate - negatives | Injuries | "Yeah, a lot of the lads, are getting…pull up on, like injuries, like little injuries like calf or groin or whatever" – A12 |
|  |  |  | "Injuries maybe, possibility of injuries. Which then affects your academics, and as A13 said, can get kicked off the program if your academics aren’t good enough. So, it’s kind of a dangerous side, especially playing rugby. Lot of injuries going on. But I think that is the whole point of strength and conditioning, it makes literally makes you healthier, makes you better, more prepared for the games. But there is always going to be a risk, however, how prepared you are." A15 |
|  |  | Body image | "I think if you already have issues around body image, or if you have issues around your ability, then that's not necessarily going to go away. Could be facilitated in the school environment and then it could carry on afterwards. So, it is possible." – C1 |
|  | Long-term - positives | Performance success | "We've had a number of kids who've come to the school who have played county cricket, so they’re playing representative cricket, some have played for the North 15s, the North 17s, so I've seen them outside of school as well as in, and we've had two who have played England under 19s which is a feather in their cap, really." – C5 |
|  |  |  | "I play for England U15 team and GB U16 team." A18 |
| Psychosocial impacts | Immediate - positives | Immediate group of friends, social support and social recognition | "The fact that they're working hard...physically working hard and we're demanding of them in the gym or on the pitches, but then the next minute they're being demanded in the classroom with a laptop. And there's also the mental fatigue as well. You know, so yes, they're physically fatigued, but also, you know, they've just played a fixture for example in Newcastle, they've got a two-hour journey, they're absolutely shattered, they're drained, physically and mentally from the game." - C3 |
|  |  |  | "I was going to say coming into a team environment, actually helped me. I know two people already existing in this school, and I am still really close with both of them. But going into a team environment and already having like, knowing that they kind of… had to be like my brothers on court as well, so then obviously we were going to make good bonds off court, and that really helped. So, I was able to get to know the team quite well and stuff. So, it helped me socially having them I could like speak too, and off that I have made a lot more new friends through my lessons, and my frees and everything. Like, because some of them are still around, as for example for me sometimes I won’t be able to get picked up to like 5:30 or 6 sometimes, so the boarders who are around I can socialise with them, and it’s just really easy, and I found it probably helped me a lot socially as well." – A17 |
|  | Immediate - negatives | Emotional, social psychological aspect of moving away from home, family and friends into a new environment | "They’re meeting new friends, so they've got a new social network. Some of the students have travelled from far away. And they’re staying in boarding which is a new challenge altogether because they're away from family for the very first time at the age of 16. So, there's the...there's the emotional and kind of social challenge of that, you know, knowing moving away from the group of friends that they've always had to come and challenge themselves in a new environment at Queen Ethelburga's with a new group of students, completely new surroundings. And all of a sudden, they're not going home to mum and dad on an evening because they're away. So, it's the...the demands on them are huge and I take my hat off to them." - C3 |
|  | Intermediate - positives | Social relationships with friendships within sport | "I also like the social side of it, it’s just a team in general, we are quite close and we all get along. But also, as a sport department, and as a whole performance program, with every other sport, it is a nice environment to be in." - A6 |
|  |  |  | "And I just think being part of the dressing room and team allows you that...the opportunity to really build friendships. And form, you know, partnerships. And then you can take that relationship off the pitch onto the pitch. And you have that togetherness on the training pitch. And the fact that, you know, you're gonna work hard for the player to the left and the player to the right. Because you've got that, you know, that good understanding and your kind of buying into the ethos of the school and the culture. So, I think that's a great...you know, developing the culture, buying into the expectations. And hopefully the students can see the long-term benefits once they do that." - C3 |
|  |  | Social status | "When you are just walking around, it’s just like, everyone just knows who you are, knows that you are a [sport] player. They’ll be like, oh that is the new scholar, that’s the new scholar, and everyone looks up to you and want to be like you. Like, to the little kids you are a role model, and they want to look up to you, so you’re setting a good image, and obviously when it comes to playing, if we are dropping numbers, then we are getting more known, and the school is getting more known, everything it getting more known." – A16 |
|  |  | Role model | “Like, to the little kids you are a role model, and they want to look up to you, so you’re setting a good image.” - A16 |
|  |  | Life skills (e.g., social skills, teamwork, ownership/responsibility, commitment, discipline, ability to take criticism, time-management, manners and maturity) | "I feel like working with other people as well like being in a team sport, you like have to have a good teamwork and stuff but then that goes to everyday life as well like in subjects, you have to work together and it all just like fits together." – A2 |
|  |  |  | "I think I have become more mature to be honest. I think I have become more mature and have become more determined to actually do something, where before kind of step back and relax." - A18 |
|  |  |  | "You know, I spoke to one, the set of parents-on-parents evening, they were like, "Don't know what you're doing with him here now, but when it comes to like weddings and things like that, he'll be stood in front of people, and he can hold conversations on an adult level. And you know, and you can just see him growing." – C6 |
|  |  |  | You have good life skills, you mature, you respectful, responsible, if you see people around you say hi, you are sociable, it’s just stuff you are wanting in everyday life. - A16 |
|  | Intermediate - negatives | Socially intense | "I guess, one thing, that might be negative. That because we such a tight group. Anything that one of us does, effects on all of us, whether that’s good or bad. But again, because we are such a tight group that kind of helps prevent those things, as much as we can." – A14 |
|  |  |  | "The day to day, I think sometimes, even its just changing the environment in which you revise in, if it’s one day in the library. Seeing the same people, seeing the same faces, seeing the same thing happen, can be intense. And again, obviously you learn a lot about certain people spending this much time with them. Sometimes it’s nice to have a breadth of fresh air to be able to change, like change, if you have been with this group as well, you can go to another. But you can’t really have that opportunity. But in general, the whole boarding environment isn’t ideal in term of that." - A18 |
|  |  | Social sacrifices with friends outside of sport | "I think it's, although I like, wouldn’t want it any different. It's sort of pushing you away from like my friends that don’t do sport because a lot of the time when I'm doing sport, they're in like their own little friend group and that’s sort of pushed away from that side of the community. But I don't really see that as a negative. I just see it's a bit like…. an aspect of it. Because although like creating a community in sports is good, it could also be a negative because it can create a divide between those who do sport and those who don't so, I think that’s like a bit of a negative." – A3 |
|  |  |  | "One of the downsides of having such a structured program, despite the fact that I like it, social life doesn’t really exist. In boarding in terms of having certain restriction, you have like curfews, things like that, you can’t just go to places and travel. So, if I was to name one negative, I would say the social side. Socially I am a better person. But in terms of opportunities to socialise there is less of that." - A18 |
|  |  | Extra-curricular sacrifices | "I think definitely this year, I have had to make a decision between these and the other extra-curricular things that I do. But, I think, I feel like I made the right choice, and I choice the right thing." - A14 |
|  |  | Hierarchy/superiority | "I think also sometimes, some people like last year there was the sixth form and they thought they were older and they can tell us what to do. This year it’s not bad like it's not like but sometimes I feel like there's like some people that feel like they don't have to do what everyone else asked to do or they can tell everyone else what, but they don't have to do it." – A3 |
| Psychological impacts | Immediate - negatives | Initial physical and mental fatigue | "The fact that they're working hard...physically working hard and we're demanding of them in the gym or on the pitches, but then the next minute they're being demanded in the classroom with a laptop. And there's also the mental fatigue as well. " - C3 |
|  | Intermediate - positives | Sport confidence | "And I think also this like session and that helped improve my confidence on [sport] because before I just struggled with the ball. I still do but I'm getting there." – A2 |
|  |  |  | "Like A17, not going to lie, at the beginning you wouldn’t of had the confidence to push someone and stuff, and now you are guarding A18 and you are putting a physical shove there, where maybe before you would of backed off. Like everyone is getting more competitive now and confident." – A16 |
|  |  | Social confidence | "I think it's made me feel more confident like when doing fitness or performance skills. It's made me feel a lot more confident but then also talking to people is building my confidence as well." – A2 |
|  |  |  | "Yeah, you see, new guys come in, who were quiet and then leave confidently." - C4 |
|  |  | Stress-relief | "The training I have with S&C, it just like stress relief, it is one of the copying mechanisms. All the work at school, you can let it out for an hour every day." - A15 |
|  |  |  | "In a public school normally, it’s just lessons and not really any sport. So, your stress may build up. Where here, you can take out some of your stress with sport, because you’re realising those endorphins. " - A16 |
|  | Intermediate - negatives | Over-confidence/ego-orientated behavior | "I suppose that I would just think about negatives there is just whether, whether their own personal ego, you know, can stand in the way of them actually getting better. When they come here got, you know, this, that and the other we've got. And being too comfortable, as well." – C6 |
|  |  | Performance pressure | "Some players do feel the pressure of being on a higher scholarship. And the fact that they need to perform to that. A bit of pressure and I also feel that…particularly for my sport there is a pressure for the team to perform because of the success that we've had previously. And I think for some of the girls…that's a big pressure. And I also think there's a pressure to be part of a franchise.” – C2 |
|  |  |  | "I think sometimes it brings pressure on. I know like it's not meant to put like, you guys aren’t there to put pressure on us but I think we do it to ourselves a lot of the time like wanting to be better and not wanting to make a fool out of ourselves sometimes in front of like for the sports teams and stuff like that so I think that part especially if you're having a bad day like said, it can just like all sort of build-up and then you go and you're like, let’s do fitness test. And it's like not necessarily the fitness test. That’s the bad thing. It's like the pressure of it that makes it the bad thing. " – A1 |
|  |  | Family pressure | "On my parents as well, they are the ones driving me here every day, paying for my new kit, paying for everything, it is a really big commitment. I do feel pressure for them to keep academics, everything balanced." – A5 |
|  |  |  | "You do feel a good amount of pressure from your parents to give back to them, and like, even your coaches, because they put so much into you, that you want too actually do well." – A8 |
|  |  | Complacency | "I think maybe, if you come into the programme, and you think it’s going to make it all happen for you, without hard work then you’re going to be a bit disappointed. Ultimately, it’s their steppingstones are put in place for the kids to use. If they don’t want to use it, they obviously won’t reach where they want to get to." – C4 |
|  |  |  | "And a lot of the time they almost think that they've made it sometimes already without having to put the work in. That every where’s not like this. And everywhere isn't catering for them. Sometimes they're gonna have to go out and find it themselves." - C2 |
|  | Long-term - positives | Resilience | "I certainly think we add an element of resilience to them… You know, that, that, that development as a young person as well." - C4 |
|  |  |  | It definitely helped me like mentally. But just sort of like resilience and so you when you do fitness, it's sort of like a lot of its mental and stuff like that so it's definitely also made me like realise more about myself and what I can do.” - A1 |
|  |  | Future confidence | "I believe that having gone through our program that they could go into any workplace and any university. And be confident to join up. So, to go to fresher’s fair and sign up on that netball sheet is…I think everybody will have the confidence to do that. And also, be confident in putting themselves out there. To do stuff to take on board like challenges and things like that. And not be daunted. I think they'll be confident to work out themselves and go to a Uni gym. And repeat what they've done. And just believe that their just as good as the next girl." – C2 |
