# Supplementary material for: A case study of the features and holistic athlete impacts of a UK sports-friendly school: Student-athlete, coach and teacher perspectives
Source: PLoS One. 2022 Nov 30;17(11):e0278401. doi: 10.1371/journal.pone.0278401 (PMC9710759; doi:10.1371/journal.pone.0278401)
Supplement: Attachment — Submitted filename: Response to Reviewers.docx [file pone.0278401.s002.docx]

PONE-D-22-07870

The features and holistic athlete impacts of a UK sports-friendly school: Student-athlete, coach and teacher perspectives.

PLOS ONE

[DATE]

Dear Dr Ender Senel

Thank you for your recent review in relation to the submission of the above article for Plos One.

Both reviews were very thorough and helpful and have allowed us to consider a number of areas, which has helped us move forward and improve the current paper.

We have addressed the majority of the comments using track changes within the paper and include a response to each of the reviewer’s comments below in relation to the changes that have been made. I hope this is useful in considering our revised submission.

I hope all the below responses and changes to the paper are to the standard expected and hope that the paper is accepted to the journal in the near future.

Yours Sincerely

Ffion Thompson

On Behalf of all co-authors

**Journal Requirements**

1. Please ensure that your manuscript meets PLOS ONE's style requirements, including those for file naming. The PLOS ONE style templates can be found at
   [https://journals.plos.org/plosone/s/file?id=wjVg/PLOSOne_formatting_sample_main_body.pdf](https://eur02.safelinks.protection.outlook.com/?url=https%3A%2F%2Fjournals.plos.org%2Fplosone%2Fs%2Ffile%3Fid%3DwjVg%2FPLOSOne_formatting_sample_main_body.pdf&data=05%7C01%7CF.Thompson9777%40student.leedsbeckett.ac.uk%7C639399ae402143873c8908da332fbe23%7Cd79a81124fbe417aa112cd0fb490d85c%7C0%7C0%7C637878577516358464%7CUnknown%7CTWFpbGZsb3d8eyJWIjoiMC4wLjAwMDAiLCJQIjoiV2luMzIiLCJBTiI6Ik1haWwiLCJXVCI6Mn0%3D%7C3000%7C%7C%7C&sdata=vAXuE4vmY81JAXTs2ZAj3nillUo6j0Vz2ocpceSlH0g%3D&reserved=0) and [https://journals.plos.org/plosone/s/file?id=ba62/PLOSOne_formatting_sample_title_authors_affiliations.pdf](https://eur02.safelinks.protection.outlook.com/?url=https%3A%2F%2Fjournals.plos.org%2Fplosone%2Fs%2Ffile%3Fid%3Dba62%2FPLOSOne_formatting_sample_title_authors_affiliations.pdf&data=05%7C01%7CF.Thompson9777%40student.leedsbeckett.ac.uk%7C639399ae402143873c8908da332fbe23%7Cd79a81124fbe417aa112cd0fb490d85c%7C0%7C0%7C637878577516358464%7CUnknown%7CTWFpbGZsb3d8eyJWIjoiMC4wLjAwMDAiLCJQIjoiV2luMzIiLCJBTiI6Ik1haWwiLCJXVCI6Mn0%3D%7C3000%7C%7C%7C&sdata=09n3xwkCULcltCREhbXh00fuX2rUVaQoq2xppwJeveo%3D&reserved=0)

**Thank you for this – we have edited the manuscript to ensure it meets PLOS ONE’s style requirements, including those for file naming.**

2. Please change "female” or "male" to "woman” or "man" as appropriate, when used as a noun (see for instance [https://apastyle.apa.org/style-grammar-guidelines/bias-free-language/gender](https://eur02.safelinks.protection.outlook.com/?url=https%3A%2F%2Fapastyle.apa.org%2Fstyle-grammar-guidelines%2Fbias-free-language%2Fgender&data=05%7C01%7CF.Thompson9777%40student.leedsbeckett.ac.uk%7C639399ae402143873c8908da332fbe23%7Cd79a81124fbe417aa112cd0fb490d85c%7C0%7C0%7C637878577516358464%7CUnknown%7CTWFpbGZsb3d8eyJWIjoiMC4wLjAwMDAiLCJQIjoiV2luMzIiLCJBTiI6Ik1haWwiLCJXVCI6Mn0%3D%7C3000%7C%7C%7C&sdata=kQeCO8NamBLCOkojvPuPdzFHbOM1hfJ6Wr%2F42aOEHpA%3D&reserved=0)).

**We have changed “female” or “male” terms to “women” or “man” as appropriate, when used as a noun.**

3. Please include your full ethics statement in the ‘Methods’ section of your manuscript file. In your statement, please include the full name of the IRB or ethics committee who approved or waived your study, as well as whether or not you obtained informed written or verbal consent. If consent was waived for your study, please include this information in your statement as well.

**Full ethics statement now stated in the ‘Methods’ section of the manuscript. This now reads (lines 468-473): “*This study was granted by university sub-ethics committee (Ref. 73464). The primary researcher contacted the participants by email and provided them with information about the research, highlighting the risks and benefits of the study. Before the interviews, written informed consent was received from all participants and parents for those under the age of 16 (n=1) received a parent information letter with a parental opt out form also provided (which was not signed).”***

4. Please include captions for your Supporting Information files at the end of your manuscript, and update any in-text citations to match accordingly. Please see our Supporting Information guidelines for more information: [http://journals.plos.org/plosone/s/supporting-information](https://eur02.safelinks.protection.outlook.com/?url=http%3A%2F%2Fjournals.plos.org%2Fplosone%2Fs%2Fsupporting-information&data=05%7C01%7CF.Thompson9777%40student.leedsbeckett.ac.uk%7C639399ae402143873c8908da332fbe23%7Cd79a81124fbe417aa112cd0fb490d85c%7C0%7C0%7C637878577516358464%7CUnknown%7CTWFpbGZsb3d8eyJWIjoiMC4wLjAwMDAiLCJQIjoiV2luMzIiLCJBTiI6Ik1haWwiLCJXVCI6Mn0%3D%7C3000%7C%7C%7C&sdata=Na28kcJ%2BKGPkCFw1bYQqrjseu3DQj5yPec2vII3%2BbPo%3D&reserved=0).

**Caption now included for supporting information files at the end of the manuscript.**

**Reviewer #1**

**General:**
The current study aims to give insights into a Dual Career environment in the UK context by interviewing both student athletes and coaches/teachers about their experience of their immediate, intermediate and long-term development. The manuscript is in general well written and structured, but rather long and in some parts are repetitive.
The current study was an interesting read and advances the literature by providing detailed description of both the benefits and drawbacks of following a dual career at sports-friendly boarding school. Please find some more specific comments below that might further enhance the quality of the manuscripts.

**Thank you for your thorough review of our manuscript and we appreciate the time taken to conduct such a review. We have considered your recommendations and made changes in the paper and have provided our responses below.**

**Abstract:**

1. It should be clear that “Salkeld High” is a fictive name or pseudonym from the beginning. I had to read until page 7 to find out about it. I would have it set in quotation marks throughout the text.

**Thank you for your comment. We have made it clear in the abstract (line 42) that “Salkeld High” is a pseudonym. This now reads: *“This study explored the perceived impacts of one sport-friendly school (pseudonym – “Salkeld High”) on holistic athlete development and the features that drove these impacts.”***

**We have also taken on board your point, and set it in quotations marks throughout the text.**

2. Sport friendly school student-athletes. Is school needed here?

**Now that “Salkeld High” has been made clear as the pseudonym in the abstract, I have changed “sport friendly school” to “Salkeld High” (line 47). This now reads (lines 43-48): *“Using a critical realist approach to thematic analysis, findings indicated a multitude of immediate, intermediate and long-term positive and negative impacts associated with academic/vocational (e.g., academic security vs. second/third choice university), athletic/physical (e.g., performance development vs. injuries), psychosocial (e.g., social skills vs. social scarifies) and psychological (e.g., sport confidence vs. performance pressure) development of “Salkeld High” student-athletes.”***

3. Keep the same order of academic/vocational; athletic/physical; psychosocial and psychological in both parts of the abstract.

**Thank you for this – the order of academic/vocational, athletic/physical, psychosocial and psychological is now consistent in both parts of the abstract.**

**Introduction:**

1. Generally, well written with relevant references. Gap in literature clearly identified (p. 5). Five gaps are addressed, will all be addressed?

**Thank you. In this study we aimed to address limitations 1, 3, 4 and 5 (not 2). Although we have provided further insight into how a sport-friendly school is operationalised in a different context to previous research, this study was an assessment of one sport-friendly school (Salkeld High), therefore, future research across different contexts within the UK and countries is warranted** **to address limitation 2. This point is addressed in section 5 (methodological reflections and future research), line 1409-1416. This read: *“Additionally, sports-friendly schools may vary in their resources, organisational structure and aims/objectives, which likely affect whether they provide benefits or contribute to school-age athletes' holistic development [7, 101]. Therefore, exploring the holistic impact of sports-friendly schools across different contexts within the UK and countries is warranted to account for the socio-cultural context and local conditions of dual-career programmes. Yet we hope that this exploration can provide a platform for further comparisons with other sports-friendly schools within the UK and different contexts.”***

2. The aim is clear and well-defined. Maybe you could mention Salked High instead of the general SfS expression in the aims.

**We have decided to keep the general expression “sports friendly school” due to the context of the study and the sample not being fully introduced until the methods section.**

**Methods:**

1. A critical realist approach was adopted, and the authors argue that a qualitative methodology enables the researcher to perceive the impacts of the students involvement in the SfS. I would argue that a case study methodology would fit the research aims better, because Stalked High is clearly your case and the student-athletes and the coaches/teachers and the first authors own experience are the informants about the case. You might want to consider re-formulating your research design.

**In this study we adopted a CR framework, which included both a qualitative and case study approach to methodology. We agree though, that the case study approach was not well outlined in our methodological approach. As such, I have tried to make this clearer in the research approach section (2.1) by adding in the following sentences (see lines 300-305): *“Furthermore, the complex and dynamic nature of sport-friendly school environments and the objective of developing a rich and detailed insight into actual existing environments called for a case study approach [11]. A case study approach is the most common, and arguable one of the most useful, form of CR research [37]. As such, this study deliberately focused on one case study to generate an in-depth, multi-faceted exploration of a complex environment in its real-life setting [38].”***

2. Context of the study (or case description, l. 155ff): It can be difficult to find the right balance to present the case and then writing in the results section about it. Please see that the information is not redundant (what does the reader need to know before the results to understand the context?).

**Thank you for this comment, we agree there is repetition between the context of study and information in the results section. As such, we have ensured the information is not redundant by drastically cutting down the information in the “context of study” section to ensure the information is what the reader needs to know before the results to understand the context of the study, without repeating any relevant information which is addressed later in the result section. This section now reads (lines 346-358): *“One sports-friendly school (pseudonym – “Salkeld High”) was selected as the case for the study based on the Morris et al. [16] definition of a sports-friendly school. The selection of “Salkeld High” was information-oriented and opportunistic. “Salkeld High” was an established and mature environment, with seven years of experience providing dual career support through a performance sport pathway embedded within a UK independent school. The school has eight performance sports as part of its performance programmes: athletics, basketball, cricket, football, hockey, netball, rugby, and swimming, targeted at year groups 7-13 (aged 12-18 years). Student-athletes accepted onto the performance sport program at “Salkeld High” pay for or receive (via scholarship) a place to study, live, and train during their secondary school years. Based on the information above, we envisioned that the stakeholders (i.e., coaches/teachers and student-athletes) embedded within “Salkeld High” would offer information-rich experiences widening the understanding holistic impacts of sports-friendly schools and how these may be connected to the school's features.”***

3. International people might not be familiar with the abbreviations of GCSE and BTEC.

**Thank you for this – this sentence in the methods has been removed, however full terms for GCSE, A-Level and BTEC have now been added later in the results section. This now reads (lines 754-756): *“… with a range of educational options in General Certificate of Secondary Education (GCSE), Advanced level qualifications (A-level), and Business and Technology Education Council (BTEC) courses.”***

4. l. 179. The first author worked (past). Is the work terminated or does it still go on?

**This primary author still works within the organisation – as such, we have changed the past tense of “worked” to “works” and "was" to "is". This now reads (lines 361-362): *“Three days a week, the primary researcher (first author) works within “Salkeld High” as the lead strength and conditioning (S&C) coach and is embedded within the context.”***

5. Participants: when reading the information about the coaches and teachers, it was not clear who was (only) a coach, or if the always have a double role. Were there any teachers interviewed that have no coaching role?

**Three out of six of the coaches had a dual role (i.e., were both a coach and a teacher). We agree this wasn’t very clear in the participant section of the methodology. As such, we have tried to make this clearer in the participant description section. This now reads (lines 440-465): *“Overall, the staff were purposefully sampled based upon coaching experience within sports-friendly schools, comprehensive knowledge of “Salkeld High” system, varied perspectives across five sports, regular contact with the student-athletes, and multiple communication links with other staff within “Salkeld High.” The staff had worked in coaching for between 11 and 42 years (mean coaching experience = 21.5 ± 11.6 years), and at “Salkeld High” for an average of 5.3 ± 3.3 years, and all had experience in more than one sports-friendly school. Furthermore, three out of the six staff members held a dual role at the school (i.e., were both a coach and a teacher). As such, the staff provide a varied perspective on holistic outcomes and a more comprehensive viewpoint on how the performance sports programme and integrated educational institute functioned.”***

**Furthermore, we have highlighted this within the abstract. This now reads (lines 39-43): *“Through a case-study approach, using five focus groups, with 19 student-athletes, and six semi-structured interviews with three coaches and three dual coach and teachers, this study explored the perceived impacts of one sport-friendly school (pseudonym – “Salkeld High”) on holistic athlete development and the features that drove these impacts.”***

6. Data analyses is well described.

**Thank you.**

7. l. 280. Figure 1 is clearly an empirical illustration of “Salked High” and should not be termed a programme theory model.

**We haven’t heard of the term “empirical illustration” – however, have taken your point on board and labelled the figure (which is now Fig 2) as a conceptual thematic map instead.**

**Results:**

1. l. 307 ff. This case description is a repetition of the “Context of the study” from the Method section. Shorten or delete.

**By cutting down the information in the “Context of study” section (see above) we have ensured there is no relevant repetition between what is said in the “Context of the study” section and the “Results” section.**

2. l. 335: Heading “Integrated Sport School Environment and Community”. Why not: “Features of Salkeld High” (as in Figure 1)?

**The overall theme/feature of Salkeld High is an “integrated sport school environment and community” and then within this are other features such as; “an integrated/structure combination of sport and education”. To clarify this in the figure (which is now Fig 2), I have removed the heading “features of Salkeld high” and made the centre heading of the circle “integrated sport school environment and community.”**

3. Just list the 6 features without elaborating here (about nr 4), you do that later in more detail.

**Point taken on board – changed to listing the 6 features without elaborating (see lines 740-746).**

4. l. 352 ff. Again, case description and repetitive. The authors are advised to go through the whole manuscripts to spot passages where the same information is given.

**By cutting down the information in the “Context of study” section we have ensured there is no relevant repetition between what is said in the “Context of the study” section and the “Results” section.**

5. In the results part, focus on the empirical qualitative material (as it is done on p. 18-27).

**Thank you for this comment, we have edited the results to focus on the empirical data throughout.**

**Discussion:**

1. The discussion is well elaborated and incorporates relevant literature.

**Thank you**

2. Methodological reflection (p. 34). Clearly, the first author has a special role and position through her involvement in the SfS under description. The potential bias is described concerning interpretation of the data. However, it should also be discussed how her position potentially impacted the data collection with student-athletes (power-relation and hierarchy) and coaches/teachers (her peers). Could they really be open and critical when talking to a member of the same institution??

**Thank you for pointing this out, we agree with your comment and have addressed this in section 5 (methodological reflections and future research). This now reads (lines 1378-1382): *“Furthermore, student-athletes and staff may have not openly expressed their concerns and opinions in front of a member of the same institution. Nevertheless, the primary author had a strong relationship with the student-athletes and staff impacting their ability (positively) to interact with her, emphasised that all data would be kept strictly confidential, and ensured participants that there were no right or wrong answers and to be open and honest.”***

3. Concerning “transition”: The term has not been introduced before in the Introduction or with references to the broad transition literature. We know from previous case studies (Henriksen et al.) that environments are dynamic and are evolving concerning their staff, members, culture, and their surroundings.

**Thank you for this point – without trying to add too much extra content (as we know this paper is long already), I have added in some additional information into the introduction to address the process of transition, as well as dynamic environments. This now reads (lines 112-115): *“Furthermore, research demonstrates the strong concurrent, interactive and reciprocal nature of transitions occurring in the sporting career (athletic transitions) and in other domains (e.g., academic, psycho-social, professional) [12, 13], as well as the complex and dynamic nature of dual career environments [11].”***

**Furthermore, we now address the point on dynamic and evolving environments further in our research approach. This now reads (lines 300-305): “Furthermore, the complex and dynamic nature of sport-friendly school environments and the objective of developing a rich and detailed insight into actual existing environments called for a case study approach [11].”**

4. Conclusions: Text and Table 1 (practical implications) is double information. Table 1 is not necessary.

**In order to ensure we are not repeating double information, we have kept the table, but reduced the information in the conclusion. This now reads (lines 1420-1453): *“To optimise the holistic development of student-athletes and in particular to remove the barriers and challenges of being involved in a sports-friendly school , they should (a) have an integrated/structured combination of sport and education (e.g., extra personal academic support and a structured academic curriculum), (b) a performance sport programme with direct sport-related practices, support services and impacts (e.g., multi-dimensional training programme and high-volume/frequency of training), (c) a strong and coherent community (e.g., align staff in academic, athletic, and psychological/psychosocial support areas and encourage community learning and promote and facilitate positive peer support), (d) a focus on holistic development on and off the pitch, (e) be aware of the intensified and challenging nature of sports-friendly school involvement (e.g., provide extra support to help student-athletes prepare for and cope with the challenges and associated changes of entering a sports-friendly school environment), and (f) collaborate with wider sporting stakeholders (e.g., in an integrated effort to balance student-athletes training schedules and develop aligned athlete-focused training plans and goals. Based on the current evidence base we have provided reasonable practical recommendations to aid the design of an appropriate sports-friendly school learning environment that drives positive holistic development (see Table 1).”***

5. Figure 1: Even though the Figure is quite loaded with information, I like the graphical summary including the development perspective/ outcomes. Should “impact” or “outcomes” be added to immediate/intermediate/long-term?
   **Thank you for the point – the term “impact” has been added to the immediate, intermediate and long-term labels in the figure (which is now Fig 2).**

I hope these comments are helpful. Best of luck in further developing this research!

**Thank you again for your thorough review of our manuscript and we appreciate the time taken to conduct such a review. We hope you are satisfied with the changes made to the paper and the points addressed.**

**Reviewer #2**

**Thank you for your thorough review of our manuscript and we appreciate the time taken to conduct such a review. We have considered your recommendations and made changes in the paper and have provided our responses below.**

Abstract

1. In the Abstract you talk about the 'Impact of sport schools features on the holistic development of student-athletes'... Would you consider reviewing the title and short title to reflect both this aim, and also to reflect the 'case study' approach? This needs to be consistent across the whole study.

**We have altered the title to reflect the ‘case study’ approach. This now reads (lines 1-2): *“A case study of the features and holistic athlete impacts of a UK sports-friendly school: Student-athlete, coach and teacher perspectives.”***

2. In Abstract, specify number of teachers, and coaches. Show the strength of your sample.

**Thank you – we have addressed this point in the abstract. This now reads (line 39-43): *“Through a case-study approach, using five focus groups, with 19 student-athletes, and six semi-structured interviews with three coaches and three dual coach and teachers, this study explored the perceived impacts of one sport-friendly school (pseudonym – “Salkeld High”) on holistic athlete development and the features that drove these impacts.”***

3. Line 31, Abstract is weak in presenting research Methods.

**Thank you for this feedback – we have presented more research methods in the abstract. This now reads (lines 39-48): *“Through a case-study approach, using five focus groups, with 19 student-athletes, and six semi-structured interviews with three coaches and three dual coach and teachers, this study explored the perceived impacts of one sport-friendly school (pseudonym – “Salkeld High”) on holistic athlete development and the features that drove these impacts. Using a critical realist approach to thematic analysis, findings indicated a multitude of immediate, intermediate and long-term positive and negative impacts associated with academic/vocational (e.g., academic security vs. second/third choice university), athletic/physical (e.g., performance development vs. injuries), psychosocial (e.g., social skills vs. social scarifies) and psychological (e.g., sport confidence vs. performance pressure) development of “Salkeld High” student-athletes.”***

Introduction/literature

1. 47-49 Need literature support.

**Literature (reference) support added.**

2. "Whole Person" - is that a quote? If yes, you need page numbers.

**This is not a quote – as such, quotation marks have been removed.**

3. page 3, 1/3.5 pages does not refer to sports and/or schools! You mention that in line 77 first. A little too late when you consider that the introduction is just 132 lines. Get the focus of the study clear earlier on.

**In our research opinion, sports school are one form of talent development programs that offers avenues for the healthy combination of sport and education (dual career) as well as holistic development. The approach to our introduction followed a specific structure (which many researchers take): starting broad at the beginning of the introduction, then gradually becoming more focused until the end of the introduction is specifically about our study. As such, in the introduction, we started broad (big picture) outlining the intensification of youth sport and how, secondly, dual careers can aid the specific challenges elite youth athletes face in balancing sport and education. This provides the rational then for the importance of holistic athletic development and how an example of a dual career-development environment that aims to cater to youth athletes' holistic development is a sports school. We then finish with what we know from other research so far about sport schools, and what we don’t know providing the rational for this study. We do feel this is the best approach to introduce the study.**

4. 82. that/which, instead of 'who permit[s]'.

**We cannot alter the grammar of this sentence as it is a direct quotation of another paper.**

5. 82. Quote is not well integrated in the sentence.

**We have attempted to better integrate the quote into the sentence. This now reads (lines 121-126): “A sports-friendly school is defined as a *“regional educational institution, who permit elite sport or align themselves with elite sport to provide academic flexibility for athletes to train and compete in their own sporting environment”* [17, p.140]. An elite sports school is defined as an *“educational institution purposefully developed for elite athletes who wish to combine their athletic and academic careers”* [17, p.140].”**

6. 82. Lines 82 to 84 you are using ' ' for direct quotes. You should be using " ". Be consistent across.

**Thank you for pointing this out, as it is a direct quote we have changed the quotations to “ ” so this is consistent across the paper.**

7. 88. Do you consider a new para before 'While exploring...'

**The original lines 88-93, have now been moved based on your comment below about order between the literature and scope.**

8. The use of unnecessary acronyms, only makes it harder for the reader. If acronyms are used to reduce word count, then perhaps you need to review your writing. Otherwise, the acronyms make it harder for readers to remember what each one of them means.

**We understand the point you are making, as such we have removed the acronym for sport school (SS), dual career (DC), sport-friendly school (SfS) and elite sports schools** **(ESS) throughout the manuscript to reduce the number of acronyms in the paper. Furthermore, full terms for GCSE, A-Level and BTEC have now been added later in the results section for clarification.**

9. 87 ISCED - in full would give more context to the readers.

**We have now written ISCED out in full. This now reads (lines 126-128): *“Both sports-friendly schools and elite sports schools are situated in lower and upper general and vocational secondary education (International Standard Classification of Education level 2–5).”***

10. in lines 88-93 you move from Literature to Scope, and then in 94 you move back to literature. Perhaps it is better if you reconsider the position of lines 88-93.

**We have taken your point on board and have relocated the original lines 88-93 to the bottom of the introduction alongside the other relevant information of the scope of the research.**

11. 94-97. It is not fully clear whether an ASS = ESS and non-ss = sfs. You need to clarify what a non-ss is. Here it very much sounds like an SFS! Unclear consistency between 94-109 and Morris' Definitions (80-86).

**Thank you for this comment, however we do not use ASS in this paper, so are unsure what you are referring too.**

12. 131. Perhaps sentence needs to become 'of an SfS in the UK and their impact on the holistic development of student-athletes, from the perspective of the student-athletes, coaches and teachers. As discussed earlier, you need to be consistent across the whole study on this.

**Thank you for this comment – sentence changed to: “*Based on the above, this study aimed to identify, explore and understand the features of an sport friendly school in the UK and their impact on the holistic development of student-athletes, from the perspective of the student-athletes, coaches and teachers.”* (lines 265-267).**

13. Paper needs better consistency in (1) Title and scope, (2) use of coaches/teachers Vs Coaches and Teachers.

**We hope the edits we have made throughout the paper have addressed point 1.**

**Regarding point 2, we have ensured consistency throughout the paper by utilising the term ‘staff’ throughout instead of coaches and teachers or coaches/teachers.**

14. Try to reduce repetitions. Certain things are repeated only few lines later.

**Thank you for this comment. We have gone through the paper and reduced any repetition as necessary.**

Methodology

1. Line 136-142 you mention critical analysis but not thematic analysis. First time you mention TA is in line 250, and you do not mention it in 'research approach'

**After providing a stronger rational for the case study approach, we personally think the introduction of thematic analysis is best situated in the data analysis section.**

2. 146-153. Focus more on data collection. Should it be moved to reduce repetition?

**Thank you for this comment, we agree there is repetition between the context of study and information in the results section. As such, we have ensured the information is not redundant by drastically cutting down the information in the “context of study” section to ensure the information is what the reader needs to know before the results to understand the context of the study, without repeating any relevant information which is addressed later in the result section. This section now reads (lines 346-358): *“One sports-friendly school (pseudonym – “Salkeld High”) was selected as the case for the study based on the Morris et al. [16] definition of a sports-friendly school. The selection of “Salkeld High” was information-oriented and opportunistic. “Salkeld High” was an established and mature environment, with seven years of experience providing dual career support through a performance sport pathway embedded within a UK independent school. The school has eight performance sports as part of its performance programmes: athletics, basketball, cricket, football, hockey, netball, rugby, and swimming, targeted at year groups 7-13 (aged 12-18 years). Student-athletes accepted onto the performance sport program at “Salkeld High” pay for or receive (via scholarship) a place to study, live, and train during their secondary school years. Based on the information above, we envisioned that the stakeholders (i.e., coaches/teachers and student-athletes) embedded within “Salkeld High” would offer information-rich experiences widening the understanding holistic impacts of sports-friendly schools and how these may be connected to the school's features.”***

3. 152. ...and to enable an in-depth...

**Sentence changed to address grammatical error – this now reads (lines 296-300): *“Employing semi-structured interviews and focus groups is compatible with a CR approach, which advocates that there must remain a degree of flexibility in how interview questions are designed to elicit information about the unique interaction of causal features in a particular context [34] and to enable an in-depth, contextualised understanding [35-36].”***

4. 158-163 Can be written better.

**We have re-written these sentences to ensure they flow and read better. This now reads (lines 348-353): *“Salkeld High” was an established and mature environment, with seven years of experience providing dual career support through a performance sport pathway embedded within a UK independent school. The school has eight performance sports as part of its performance programmes: athletics, basketball, cricket, football, hockey, netball, rugby, and swimming, targeted at year groups 7-13 (aged 12-18 years).***

5. 164. Athletics and swimming wouldnt' probably cover tactics. Or would they?

**This is an interesting question. From chatting to swimming and athletics coaches they would argue that tactical aspects such as pacing in races, judging when to attack in a long-distance event, what height to come in during a field jumping event etc… are important aspects of their sport.**

6. 179-180 Presents an issue of confidentiality. We know the author, so if one does a search for the author one would probably discover Salked High's real name. If I am right, I would say that Salked High in reality is Queen Ethelburga’s (QE) College... if this is yours - <https://www.leedsbeckett.ac.uk/blogs/carnegie-xchange/2021/04/the-impacts-of-sport-schools-on-holistic-athlete-development-ffion-thompson/>

**We understand the issue you have raised. However, positionality is integral to the process of qualitative research, which cannot be ignored. It is important (and some deem essential) for researchers to identify and clearly articulate their positionality in respect of the project being undertaken (Gary and Holmes 2020). Positionality is an important consideration because it not only directly influences how the research is carried out but also determines the prevailing outcomes and results (Rowe, 2014). Therefore, it is important that the researcher declares their positionality in order to ensure that acknowledgment and allowances are made by the researcher to locate their views, values, and beliefs about the research design, conduct, and output(s) (Gary and Holmes 2020). As such, in this study we deem it important for the primary research to declare their positionality.**

**In addition to school and university ethical procedures/guidelines, the research was governed by ethical principles provided by professional and regulatory bodies. (BPS code of ethics and conduct:** [**https://www.bps.org.uk/news-and-policy/bps-code-ethics-and-conduct**](https://www.bps.org.uk/news-and-policy/bps-code-ethics-and-conduct)**. Health and care professions council (HCPC) Standards of conduct, performance and ethics:** [**https://www.hcpc-uk.org/aboutregistration/standards/standardsofconductperformanceandethics/**](https://www.hcpc-uk.org/aboutregistration/standards/standardsofconductperformanceandethics/)**.)**

**Within the gatekeeper and participant consent forms the risk of confidentiality were clearly outlined to all individuals: *“Although identities will remain anonymous and that an identifiable information about individuals or settings mentioned in an interview will not be disclosed, there remains a small possibility that someone could recognize your organisation as a participant in this project.”* We also got clearance from the school principal for the anonymised data included within this research project to be used in final reports, publications and presented during conferences.**

**Therefore, overall, although there is a risk of the institution being identified, procedures are put in place to protect the identity of the individual participants within that institution. For example, although quotes were included in the results, no details were provided as to the sport, age, or subject group of participants (i.e., coaches/teachers were just be referred to as staff and young people referred to as athletes). Furthermore, we have taken out relevant information about the director of sport and sports of the coaches/teachers to protect their anonymity further. Finally, by not declaring the year the data was collected, readers are not able to identify any individuals from the information provided.**

7. 195 and 205. Selection Criteria and actual selection don't fully agree. In line 195 you say that participants had to be... the head coach of one of the programmes. In line 205 you say that one was director of sport. This raises questions. Why?

**Due to the issue raised above about confidentiality, we have taken out the part outlining the director of sport involvement in the participant information section (2.4). As a result, we have also slightly altered the selection criteria. This now reads (see lines 429-431): *“Participants had to meet one of the following criteria: (a) be a staff member at “Salkeld High” or, (b) participate as an athlete on “Salkeld High’s” performance sport program.”***

8. 206. 'three of them being teachers' needs to be explained better (consistency with line 212 is important).

**Three out of six of the coaches had a dual role (i.e., were both a coach and a teacher). We agree this wasn’t very clear in the participant section of the methodology. As such, we have tried to make this clearer in the participant description section. This now reads (lines 440-465): *“Overall, the staff were purposefully sampled based upon coaching experience within sports-friendly schools, comprehensive knowledge of “Salkeld High” system, varied perspectives across five sports, regular contact with the student-athletes, and multiple communication links with other staff within “Salkeld High.” The staff had worked in coaching for between 11 and 42 years (mean coaching experience = 21.5 ± 11.6 years), and at “Salkeld High” for an average of 5.3 ± 3.3 years, and all had experience in more than one sports-friendly school. Furthermore, three out of the six staff members held a dual role at the school (i.e., were both a coach and a teacher). As such, the staff provide a varied perspective on holistic outcomes and a more comprehensive viewpoint on how the performance sports programme and integrated educational institute functioned.”***

**Furthermore, we have highlighted this within the abstract. This now reads (lines 39-43): *“Through a case-study approach, using five focus groups, with 19 student-athletes, and six semi-structured interviews with three coaches and three dual coach and teachers, this study explored the perceived impacts of one sport-friendly school (pseudonym – “Salkeld High”) on holistic athlete development and the features that drove these impacts.”***

9. Thematic analysis is not supported by any reference, nor it is clear which process of thematic analysis was applied. Clarke and Braun?

**Thank you for this point – we have now supported the thematic analysis process with references and clearly outline which process of thematic analysis was applied. This now reads (lines 530-537): *“This study aimed to provide an exploratory (i.e., seeking to describe a phenomena) and an explanatory (i.e., seeking to explain the causes of phenomena) approach to research [40]. As such in analysing the data, Fryers’ [42] five-step critical realist approach to TA was aligned with Wiltshire and Ronkainen [43] three types of themes (experiential, inferential and dispositional), represented in Fig 1. This approach required data-driven coding, deductive thinking and inductive thinking (which are currently used in other approaches to TA [44]) as well as abductive and retroductive thinking (which are advocated in realist methodology [e.g., 45-46]).”***

**Please refer to section 2.6 (‘data analysis’) for further explanation of the thematic analysis process adopted.**

10. The concept of trustworthiness needs to be underlined, especially to clarify how data led, to the findings in a trustworthy manner, and how themes emerged from that same process.

**We have now added a “Markers of quality” (section 2.7). The term ‘trustworthiness’ is not a word used in reference to Maxwell. What is traditionally captured under trustworthiness is part of the adequacy and plausibility of the research. Therefore, in this study we have outlined how in the following Ronkainen & Wiltshire's [45] realist suggestions for qualitative research in sport and exercise psychology and Maxwell's [37, 46] validity criteria, we have established confidence in our findings' adequacy, plausibility, and utility interpretations.**

**This now reads (lines 590-651): *“In line with our CR approach, we judged the adequacy, plausibility and utility of our explanations as the main criteria to judge the study against [47]. Following the recommendations of Ronkainen & Wiltshire [47], we drew upon Maxwell's [38, 48] validity criteria, to establish confidence in the finding’s adequacy, plausibility and utility. Adequacy was ensured through recording and precise transcription of all interviews and focus groups, with transcripts thoroughly checked for accuracy against these recordings. Secondly, we foregrounded the voice of the participants and provide quotations throughout the results to provide evidence of empirical justification. In terms of plausibility, frequent peer-debriefing and critical reflections sessions occurred between the researcher and her supervisory team, where the first author's initial interpretations and proposed explanations were reviewed, discussed and challenged [49]. These discussions led to regrouping of subthemes into higher order themes (e.g., sporting and wider community under one main theme – integrated environment and community), and moving from presenting features largely descriptively (listing themes) to a more narrative explanation that captures how these features produce impacts. Furthermore, protocols were put in place to ensure honesty in informants when contributing data (e.g., right to withdraw and reiteration of confidentiality). Finally, regarding utility, we provided practical recommendations and interpretations to guide practical actions in the real-world.”***

Results

1. I would rather use the term 'FINDINGS' instead of 'RESULTS'. But feel free to decide.

**Section 3 has been re-labelled to “Findings”**

2. If the last figure on the last page is Figure 1, it needs a clear caption with numbering.

**Apologies for this. Now the figure (which is now Fig 2) has a clear caption with numbering: Figure 2. A conceptual thematic map of “Salkeld High”**

**I have also clearly outlined in the text now where this figure should be located in the paper (line 661).**

3. 320-321. "So I think..." Sentence is not conclusive.

**To support this quote, we have now provided more context for the reader, in relation to what question the response was answering. This now reads (lines 706-710): This is demonstrated by Staff 6, when asked if the skills student-athletes developed from the sport program could be transferred to other aspects of life; *“That culture that set in, you know, the coaches push them, don't get me wrong, you know, there's, there's no hiding places in many sessions. So, I think that resilience to keep going when it does get tough.”***

**Furthermore, to make it more conclusive, we have added an additional athlete quote as well (see lines 710-713):**

**This development of resilience was further supported by athlete 1: *“It definitely helped me like mentally. But just sort of like resilience and so you when you do fitness, it's sort of like a lot of its mental and stuff like that so it's definitely also made me like realise more about myself and what I can do.”***

4. Ex 318-321. Participants' direct quotes need to be better integrated.

**We believe, we have now better integrated the direct quotes, referred to above (lines 706-713).**

5. 321-325. It is an important deep analysis of the implications of the model. Set it in a more 'visual' manner, perhaps in a section on its own?

**We have moved this passage of literature to it’s own separate paragraph (lines 741-720).**

6. 326-327. Better in Limitations?

**We have decided to keep this point where it is. We do elaborate on this point in further detail in the limitation section (section 5). However, we do think it is important when explaining the figure, to explain why there is a lack of long-term positive and negative impacts. Furthermore, we think it is important to make the readers aware of this point early on in the results section, before we elaborate on the findings in more detail, as it provides context/rational to why there isn’t much detail in the findings section on long-term impacts.**

Discussion and Conclusion

1. 816-818. Perhaps better to underline in Methodology?

**We appreciate your comment, however as a research group, we personally think it is important to address this in the methodological reflection section. So, to avoid repetition within the text, we have decided not to underline the original 816-818 lines in the methodology section.**

Important Points

1. This study needs to clearly indicate the 'Themes', 'Sub Themes, and categories emerging from the data analysis. There seems to be limited coherence in the process of data analysis, presentation of 'results' and 'conclusion'.

**Thank you for this insight. In order to address the points above, we made the following changes:**

1. **Within the data analysis section (2.6), we have outlined the steps of our thematic analysis approach, more clearly, highlighting how we went about analysing out data, the exact steps we took, and how we came to the results presented in the “findings section” and “figure 2”. This should provide clarity of the codes and generated from the data analysis, and provide more coherence between the process of data analysis and presentation of ‘findings.’**

**This now reads (lines 530-575):**

***“This study aimed to provide an exploratory (i.e., seeking to describe a phenomena) and an explanatory (i.e., seeking to explain the causes of phenomena) approach to research [40]. As such in analysing the data, Fryers’ [42] five-step critical realist approach to TA was aligned with Wiltshire and Ronkainen [43] three types of themes (experiential, inferential and dispositional), represented in Fig 1. This approach required data-driven coding, deductive thinking and inductive thinking (which are currently used in other approaches to TA [44]) as well as abductive and retroductive thinking (which are advocated in realist methodology [e.g., 45-46]).***

***As part of the first stage of thematic analysis, the primary author clearly outlined and refined the research aims and objectives (i.e., to identify, explore and understand the features of a sport friendly school in the UK and their impact on the holistic development of student-athletes). In the second stage, the primary author immersed themselves in the data, by listening to the audio recordings, undertaking verbatim transcription, reading and re-reading the data to familiarise themselves with the findings and making notes on the initial thoughts and questions. Following familiarization, stage three consisted of applying, developing and reviewing codes (step 3; [42]) to create experiential themes [43]. Experiential themes describe participants’ subjective viewpoints and experiences as they are evident in the data [43]. In this study, data on holistic athlete impacts were deductively categorised within four higher-order themes derived from the Holistic Athletic Career model (i.e., academic/vocational, athletic/physical, psychosocial and psychological impacts) with raw data responses inductively coded into more meaningful middle and lower-order sub-themes (e.g., life skills were coded into lower order sub-themes such as ownership/responsibility, commitment). Successively, the holistic athlete impacts were categorised under immediate, intermediate and long-term impacts. Immediate impacts referred to impacts experienced by the student-athletes as they transitioned into “Salkeld High”. Intermediate denoted to impacts experienced by student-athletes during their time at “Salkeld High” and long-term impacts referred to impacts student-athletes experienced after leaving the “Salkeld High” environment.***

***Following the development of codes, step 4 entailed developing themes (step 4; [42]) to create dispositional themes [43]. Dispositional themes entail thinking about the mechanisms that have real causal influence on the world [43]. The primary researcher interpreted the data to provide more causal explanations and narrative, outlining how particular features of “Salkeld High” produce the experiences and events evident in the data and codes (e.g., holistic impacts). Finally, within stage five [42], we developed a conceptual thematic map of “Salkeld High” (Fig 2). The diagrams provide a conceptual visualisation, that combines the dispositional themes (e.g., integrated sport school environment and community), with the experiences (experimental themes) of the participants in the research (e.g., immediate, intermediate, and long-term, positive and negative holistic impacts).”***

2. **To further provide more clarity of codes/themes from the data analysis, and provide more coherence between the process of data analysis and presentation of ‘findings’ and figure 2, we have developed a figure (figure 1) outlining the Thematic Analysis Process more clearly, and how we progressed from the first stage of experiential coding (i.e., 4 big headings according to Wylleman, with immediate, intermediate and long-term impacts) to the development of dispositional themes, providing more causal explanations and narrative, outlining how particular features of “Salkeld High” produce the experiences and events we saw in our data and codes (e.g., holistic impacts). Then finally, how we subsequently developed a conceptual thematic map of “Salkeld High” (Figure 2), which provides a conceptual visualisation, that combines the dispositional themes (e.g., integrated sport school environment and community), with the experiences (experimental themes) of the participants in the research (e.g., immediate, intermediate, and long-term, positive and negative holistic impacts). The figure headings align to what we presented in the findings section.**
3. **Finally, we have edited the conclusion to now highlight/reflect more coherently with the themes developed within the paper.**
2. For a Thematic Analysis Approach, it would be better to use a categorisation chart to show how the data led to the themes, sub themes and categories emerging from the data.

**Taking your point on board, we have provided a figure (figure 1) outlining the Thematic Analysis Process more clearly, and how we progressed from the first stage of experiential coding (i.e., 4 big headings according to Wylleman, with immediate, intermediate and long-term) to the development of dispositional themes, providing more causal explanations and narrative, outlining how particular features of “Salkeld High” produce the experiences and events we saw in our data and codes (e.g., holistic impacts). Then finally, how we subsequently developed a conceptual thematic map of “Salkeld High” (Figure 2), which provides a conceptual visualisation, that combines the dispositional themes (e.g., integrated sport school environment and community), with the experiences (experimental themes) of the participants in the research (e.g., immediate, intermediate, and long-term, positive and negative holistic impacts). The figure headings align to what we presented in the findings section.**

3. Coherence across the subtitles in the 'results' section and the conclusion / figure 1 may aid in having further clarity as to what the main findings are. Hence, perhaps coherence between the emerging themes (sub themes, categories) and the sections within the results' section would assist in the requested coherence.

**Thank you for this point – I have made the subtitles in the ‘findings’ section more coherent with the conclusion and figure 2.**

1) **In the conclusion, this now reads (lines 1420-1453): *“To optimise the holistic development of student-athletes and in particular to remove the barriers and challenges of being involved in a sports-friendly school , they should (a) have an integrated/structured combination of sport and education (e.g., extra personal academic support and a structured academic curriculum), (b) a performance sport programme with direct sport-related practices, support services and impacts (e.g., multi-dimensional training programme and high-volume/frequency of training), (c) a strong and coherent community (e.g., align staff in academic, athletic, and psychological/psychosocial support areas and encourage community learning and promote and facilitate positive peer support), (d) a focus on holistic development on and off the pitch, (e) be aware of the intensified and challenging nature of sports-friendly school involvement (e.g., provide extra support to help student-athletes prepare for and cope with the challenges and associated changes of entering a sports-friendly school environment), and (f) collaborate with wider sporting stakeholders (e.g., in an integrated effort to balance student-athletes training schedules and develop aligned athlete-focused training plans and goals. Based on the current evidence base we have provided reasonable practical recommendations to aid the design of an appropriate sports-friendly school learning environment that drives positive holistic development (see Table 1).”***
2) **The figure headings (of figure 2) now align to what we presented in the findings section.**
4. Table 1 is an important figure, but seems to be skipping an important step, that of the 'clarity' of the emerging themes.

**Hopefully we have addressed this issue with the comments above.**

5. It is not exactly clear what the figure on the last page is presenting - it has no caption to support its position either.

**Apologies for this. The figure now has a clear caption with numbering: Figure 2. A conceptual thematic map of “Salkeld High”**
