# Supplementary material for: A case study of the features and holistic athlete impacts of a UK sports-friendly school: Student-athlete, coach and teacher perspectives
Source: PLoS One. 2022 Nov 30;17(11):e0278401. doi: 10.1371/journal.pone.0278401 (PMC9710759; doi:10.1371/journal.pone.0278401)
Supplement: Attachment — Submitted filename: Response to Reviewers (2.0).docx [file pone.0278401.s003.docx]

PONE-D-22-07870

The features and holistic athlete impacts of a UK sports-friendly school: Student-athlete, coach and teacher perspectives.

PLOS ONE

28^th^ September 2022

Dear Dr Giancarlo Condello

Thank you for your recent review in relation to the submission of the above article for Plos One.

The review was very thorough and helpful, and has allowed us to consider a number of areas to help us move forward and improve the current paper.

We have addressed the majority of the comments using track changes within the paper and include a response to each of the reviewer’s comments below in relation to the changes that have been made. I hope this is useful in considering our revised submission.

I hope all the below responses and changes to the paper are to the standard expected and hope that the paper is accepted to the journal in the near future.

Yours Sincerely

Ffion Thompson

On Behalf of all co-authors

**Journal Requirements**

1. Please review your reference list to ensure that it is complete and correct. If you have cited papers that have been retracted, please include the rationale for doing so in the manuscript text, or remove these references and replace them with relevant current references. Any changes to the reference list should be mentioned in the rebuttal letter that accompanies your revised manuscript. If you need to cite a retracted article, indicate the article’s retracted status in the References list and also include a citation and full reference for the retraction notice.

**After thorough review, no papers within this manuscript have been retracted. As such, no references were removed from the paper. However, during this process we found two duplicate references, which we have been addressed. We are confident now that the revised reference list is fully completed and correct.**

**Reviewer #1**

**General:**

I congratulate the authors for significantly improving the manuscript and for doing a very thorough job by revising the text based on my and the other reviewer’s comments. After reading the new version, I have a few comments or suggestions that the authors may take on board for the next revision.

**Thank you for your thorough review of our manuscript and we appreciate the time taken to conduct such a review. We have considered your recommendations and made changes in the paper and have provided our responses below.**

**Abstract:**

1. Well described and improved

**Thank you very much.**

**Introduction:**

1. Generally, well written with relevant references.

**Thank you very much.**

2. l. 123 “limited research evaluates how features affect athlete impact” is not clear.

**We have elaborated on this sentence to hopefully make it clearer. This now reads (line 157-160): *“limited research evaluates how features affect athlete impacts (i.e., it is not possible within the current literature to establish a rigorous causal relationship between the characteristics and features of sports school and holistic athlete impacts)”***

3. l. 127. Kuettel, Christensen, Zysko & Hansen (2020) compared DC environments across Europe and emphasized the different DC mindsets depending on the cultural context.

**Thank you for referring us to this paper. I have included it as a reference to help support the point that it is important to understand environments within a particular context (in this case the UK) due to the different DC mindsets depending on the cultural context.**

4. l. 128-129. The same information is given in line 140-142. And again in 146-147. I believe it is most appropriate to state the aims just before the Method section.

**Thank you for this point. To avoid repetition and in line with your advice to state the aims just before the method section, what was previously l. 128-129 and l. 146-147 have been removed. The aim is mentioned on lines 175-177.**

**Methods:**

1. Critical realism is better explained and integrated in the project’s description.

**Thank you very much.**

2. Participants are well explained. Data analyses and collection are well explained. It could be considered to first explain the collection part and then the analysis strategy and steps.

**We believe that we have stated the data collection part of the methodology first (section 2.5) followed by the data analysis strategy and steps (section 2.6). But please let us know if we are misunderstanding your comment.**

3. l. 276 the author immersed herself (singular)

**Changed to herself (singular). This now reads (line 284): *“the primary author immersed herself in the data.”***

4. l. 306 “Markers of Quality” heading is not very meaningful. Maybe add something with “research rigor”?

**Thank you for your suggestion – heading has been changed to *“establishing research rigor”* (line 348).**

**Findings:**

1. The first part of the findings make sense (summarizing the results with Figure 2). However, the part starting from 347 until 367 seems somehow misplaced (is it needed at all?). I also do not recall that resilience was a topic described earlier or later in the discussion.

**Thank you for the suggestion. We agree the lines suggested are not necessary, and as such have been removed from the manuscript.**

2. The part 355-359 belongs more to the discussion part (it is repeated shortly in the methodological reflections).

**Thank you for this suggestion. What was previously lines 355-359 how now been moved to the methodological reflection part of the discussion. See lines 1029-1038.**

3. Heading numbers: Integrated Sport Environment and Community is labelled 3.1. but I could not find any heading 3.2. Maybe it is more meaningful to label the six themes of your thematic analyses with 3.1 An integrated/structured combination…; 3.2. A performance program…; 3.3. A strong and coherent community; 3.4 ….

**Thank you for pointing this out. We have now ensured that all heading numbers are labelled correctly, and have labelled the six themes like you have suggested (e.g., 3.1 An integrated/structured combination…; 3.2. A performance program…; 3.3. A strong and coherent community; 3.4 ….).**

**Discussion:**

1. The discussion is well elaborated and incorporates relevant literature. Since you present a new conceptual map for exploring DC environments (Figure 2), it would be nice to read more about how your framework is different/better than previous frameworks described in the literature (Henriksen´s ATDE/ESF or DCDE/DC-ESF)

**It wasn’t the aim of this paper to produce a model. The conceptual map was developed to present a summary of our findings in a concise and visual manner. Although we agree it would be interesting to explore how this figure compared to other frameworks, we respectfully do not think it is necessary within the scope of this paper. We hope that in the discussion we have offered sufficient comparison to existing literature to place our findings in relation to previous findings and existing frameworks.**

2. Smith & McGannon reference is double (49 and 98)

**Thank you for pointing this out – we have addressed this issue, and ensured all reference formatting is correct with no duplicates.**

I hope these comments are helpful. Best of luck in further developing this research!

**Thank you for your thorough review of our manuscript and we appreciate the time taken to conduct such a review. We hope you are happy with the above responses and changes to the paper.**
